# Chemosensitization of leukemia cells through inhibition of NAMPT

**DOI:** 10.1186/2049-3002-2-S1-P24

**Published:** 2014-05-28

**Authors:** Theresa Gorski, Stefanie Petzold-Quinque, Susanne Schuster, Melanie Penke, Sandy Richter, Wieland Kiess, Antje Garten

**Affiliations:** 1 Center for Pediatric Research Leipzig (CPL), University Hospital for Children and Adolescents, University of Leipzig, Leipzig, Germany

## Background

NAMPT (Nicotinamide phosphoribosyltransferase) catalyzes the rate-limiting step in the NAD-biosynthesis from nicotinamide and regulates the activity of NAD-dependent enzymes. Cancer cells are highly dependent on NAD for energy and DNA repair processes and are expected to be more susceptible to the inhibition of NAD synthesis than non-transformed cells. Can inhibition of NAMPT by FK866 sensitize leukemia cells for chemotherapeutic agents?

## Materials and methods

Viability was measured using WST-1 assay. Cell death was analysed in Jurkat and Molt-4 cells by PI staining. NAD levels were measured using a colorimetric NAD/NADH Assay or HPLC. NAMPT activity was measured in leukemia cell lines and PBMCs (peripheral blood mononuclear cell) using radioactively labelled ^14^C-nicotinamide.

## Results

NAMPT expression and enzymatic activity were significantly higher in leukemia cell lines compared to normal PBMCs. Incubation with FK866 [10nM] for 24h reduced NAMPT activity by 91.1±3.6% in Jurkat cells and by 97.8±1.2% in Molt-4 cells. NAD levels were reduced by FK866 by 83.9±1.0% (Jurkat) or 79.2±2.8% (Molt-4). The combination of etoposide and FK866 caused increased cell death compared to each substance alone. In contrast, combining FK866 and methotrexate or doxorubicin showed no increased effect on cell death. Etoposide decreased the expression of the NAD-dependent deacetylase SIRTUIN1 (SIRT1). The acetylation of the SIRT1 target p53 was increased after stimulation with etoposide and was further enhanced after combining etoposide with FK866. Concomitantly, the transcriptional activity of p53 was increased as shown by an increased expression of p21.

## Conclusion

The combination of etoposide and FK866 caused increased cell death and induced acetylation and transcriptional activity of p53. Combining FK866 and etoposide could therefore be a novel therapeutic strategy to enhance the efficacy of etoposide against leukemia cells.

